# The Risks We Dread: A Social Circle Account

**DOI:** 10.1371/journal.pone.0032837

**Published:** 2012-04-11

**Authors:** Mirta Galesic, Rocio Garcia-Retamero

**Affiliations:** 1 Center for Adaptive Behavior and Cognition, Max Planck Institute for Human Development, Berlin, Germany; 2 Department of Experimental Psychology, University of Granada, Spain; University of Utah, United States of America

## Abstract

What makes some risks dreadful? We propose that people are particularly sensitive to threats that could kill the number of people that is similar to the size of a typical human social circle. Although there is some variability in reported sizes of social circles, active contact rarely seems to be maintained with more than about 100 people. The loss of this immediate social group may have had survival consequences in the past and still causes great distress to people today. Therefore we hypothesize that risks that threaten a much larger number of people (e.g., 1000) will not be dreaded more than those that threaten to kill “only” the number of people typical for social circles. We found support for this hypothesis in 9 experiments using different risk scenarios, measurements of fear, and samples from different countries. Fear of risks killing 100 people was higher than fear of risks killing 10 people, but there was no difference in fear of risks killing 100 or 1000 people (Experiments 1–4, 7–9). Also in support of the hypothesis, the median number of deaths that would cause maximum level of fear was 100 (Experiments 5 and 6). These results are not a consequence of lack of differentiation between the numbers 100 and 1000 (Experiments 7 and 8), and are different from the phenomenon of “psychophysical numbing” that occurs in the context of altruistic behavior towards members of other communities rather than in the context of threat to one's own community (Experiment 9). We discuss several possible explanations of these findings. Our results stress the importance of considering social environments when studying people's understanding of and reactions to risks.

## Introduction

It is common for people to dread some risks but not others: They tend to be very afraid of epidemic diseases, nuclear power plant failures, and plane accidents but are relatively unconcerned about some highly frequent and deadly events, such as traffic crashes, household accidents, and medical errors. One key distinction of dreadful risks seems to be their potential for catastrophic consequences [1], threatening to kill a large number of people within a short period of time [2]. In this paper, we propose that people may be particularly sensitive to risks that could wipe out the number of people that corresponds to the size of a typical *social circle*–family, friends, colleagues, and others that people interact with on a regular basis [3], [4].

What is the size of a typical social circle? In hunter-gatherer societies–typical for most of human evolutionary history–people frequently lived in bands of 20 to 50 individuals [5], [6]. Several adjacent bands often maintained regular contact for collaborative activities or ceremonial purposes, creating a local community of about 100 to 200 individuals [7]. In modern societies, the same groupings appear in personal and professional social networks [8]. For example, in a recent study of a Dutch population (Galesic, Olsson, Rieskamp, in preparation), participants maintained regular face-to-face contact with a median of 50 individuals (interquartile range: 20–100 people). The number of individuals with whom people are in regular–but not necessarily face-to-face–contact is somewhat higher: Participants in a study in the United Kingdom sent Christmas cards to a median of 138 people (interquartile range: 88–213 people) [9]. Similarly, the average number of contacts that people have at a large social networking site is 130 [10]. Larger social circles are relatively rare, possibly because humans have a cognitive limit allowing them to maintain stable relationships with at most 150 individuals [8]. In sum, although there is a large variability in reported sizes of social circles, active contact seems to be rarely maintained with more than 100 to 200 people.

Why would people be especially afraid of risks that could kill roughly the number of people that corresponds to the size of a typical social circle? One possibility is that the fear developed in our evolutionary history. Given the very low population densities throughout most of the Paleolithic–about 1 person per 10 square miles [11]–losing one's community might have implied being alone for days or longer before encountering another group. This would have been a serious threat to one's fitness, as being in a group reduces predation risk, helps with finding food and hunting, and increases survival chances when injured [12]–[15]. People who were more afraid of risks that could kill their whole group might have been more likely to engage in behaviors that helped the group to escape or reduce the impact of the dreaded risk (e.g., avoiding contaminants that could lead to an epidemic infection, urging the group to evacuate a dangerous area, trying to prevent the risk, or helping others who were affected), thus saving their group and with it increasing their own fitness. Therefore, although today population densities are many times higher than in the Paleolithic and the next human being is typically just minutes away, people may still have a tendency to be particularly sensitive to risks that could destroy a typical social circle.

If it is true that people dread risks that threaten to kill roughly the number of people that corresponds to the size of a typical social circle, then risks that threaten a much larger number of people should not be dreaded more. To investigate this idea, we conducted a series of 9 experiments. In Experiments 1 to 4, we tested a very specific hypothesis: Risks that kill 100 people would be feared *more* than those that kill 10 people, but risks that kill 1000 people would *not* be feared more than the risks that kill 100 people. That is, we expected this pattern of results rather than a monotonic increase of fear with increase in group size. The rationale is that (a) risks that kill 10 people are insufficient to wipe out everyone in a typical social circle and, therefore, will cause moderate fear, (b) risks that kill 100 people suffice to eliminate most of a typical social circle and hence will produce more fear than risks that kills 10 people, and (c) risks that kill 1000 people cannot cause much additional damage to one's social circle than those that kill 100 people and hence will not be feared more. In Experiments 5 and 6, we used a different methodology to directly test the hypothesis that dread peaks when a risk threatens to kill the number of people that corresponds to the size of a typical social circle. In Experiments 7 and 8, we ruled out an alternative explanation of the results. Finally, in Experiment 9, we show that this pattern of results is not the same as the “psychophysical numbing” phenomenon.

## Experiments 1–4

### Method

#### Ethics statement

All experiments in this paper were approved by the Ethics Committee at the University of Granada. Participants had to confirm that they have read and agreed to a written paper-based (Experiments 1 and 7) or electronic (other experiments) informed consent form before starting the study. Three participants in Experiment 2 and one in Experiment 8 reported to be 17 years old and they were excluded from the analysis.

#### Participants

Experiment 1 was conducted in January 2009 with *n* = 83 undergraduates (25% men, age 18–37 years) in the lab at the University of Granada in Spain. Experiment 2 was conducted in February 2009 with *n* = 44 participants (30% men; age 18–63 years), recruited via two Web sites that linked to psychological experiments (http://psych.hanover.edu/Research/exponnet.html and http://genpsylab-wexlist.unizh.ch/). Two thirds [29] of the participants in Experiment 2 were from the United States while a third lived in other countries (U.K., Germany, Australia, or others). Participants in Experiments 3 (*n* = 30, 50% men, age 18–69 years) and 4 (*n* = 30, 35% men, age 22–67 years) were recruited via the Web marketplace Amazon Mechanical Turk, which provides a convenient pool for conducting some types of behavioral research [16]; all participants reported being from the United States. Experiments 3 and 4 were conducted in February 2011.

#### Materials and Procedure

In Experiments 1 to 4, participants read a brief description of a scenario involving risks that threaten to kill a specific number of people. In Experiment 1 and 2, the risk was described as “an unknown, deadly disease affecting your town.” In Experiment 3, the risk was described as “an accident in a factory near your town, resulting in a release of poisonous fumes into the air.” Finally, in Experiment 4, the risk was described as “an earthquake that struck a part of your town.” In each experiment, randomly chosen groups of participants were told that the risk was forecasted to kill 10, 100, or 1000 people in their town within a week. Participants were then asked to rate how much they would be afraid of that risk on an 11-point scale ranging from 0 (“not at all”) to 10 (“very much”). As a control, we further asked participants to estimate the size of the town they lived in at that time and to report their age and sex.

### Results

**Figure 1:**
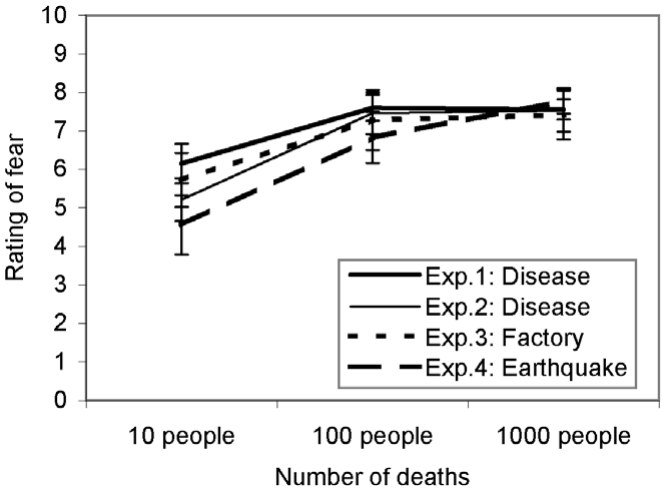
Ratings of fear of risk killing 10, 100, or 1000 people, obtained in Experiments 1–4. Error bars represent ±1 standard error.

In line with our hypothesis, in all four experiments there was a significant increase in fear of risks killing 10 vs. 100 people, but no significant difference in fear of risks killing 100 vs. 1000 people (see Figure 1). For example, in Experiment 1 the mean difference (±SE) in ratings of fear of a disease killing 10 and 100 people was 1.4±0.55, *d* = 0.64, whereas there was a difference of −0.04±0.54, *d* = 0.03, in ratings of fear of diseases killing 100 and 1000 people. In Experiment 3, the mean difference in ratings of fear of factory accidents killing 10 and 100 people as a result of the release of poisonous fumes into the air was 1.6±1.11, *d* = 0.71, whereas there was a difference of 0.13±1.02, *d* = 0.06, in ratings of fear of factory accidents killing 100 and 1000 people. Similar results were obtained in Experiments 2 and 4 (Figure 1). There were no significant interactions of the number of deaths and sex, age, or town size of the participants. There was a tendency for women to give higher ratings of fear than men, but it did not interact with the pattern of results reported above.

## Experiments 5 and 6

To test if the results in Experiments 1 through 4 are partially due to some methodological artifact (e.g. scale-end effects that deflate high ratings [17]), in Experiments 5 and 6 we employed a different method. In particular, we asked participants to estimate the number of deaths that would make them feel different levels of fear in the scenarios used in the previous experiments (i.e., disease, factory accident, and earthquake). In addition, in Experiment 6 we asked participants to estimate the number of individuals in their social circle to test if our assumption about the size of a typical social circle is correct. If dread is indeed related to the risk of a typical social circle being wiped out, then, on the group level, the average number of deaths needed to feel the maximum fear should correspond to the size of a typical social circle. In addition, individual differences in the size of social circles may affect the results.

### Method

#### Participants

Participants in Experiments 5 (*n* = 92, 44% men, age 20–64 years) and 6 (*n* = 86, 45% men, age 18–72 years) were recruited via the Web marketplace Amazon Mechanical Turk. Experiment 5 was conducted in February 2011 and Experiment 6 in March 2011. All participants reported to be from the United States.

#### Materials and Procedure

Participants estimated the number of deaths that would need to occur in order for them to feel five different levels of fear ranging from “not at all afraid” to “very afraid.” Random subgroups of participants were asked to imagine different risk scenarios, all already used in Experiments 1 to 4: disease, factory accident, and earthquake. In addition, participants in Experiment 6 were asked to estimate the size of their social circle using the *summation method* [18]. In this method, participants report the size of different groups within their social circle, defined as the number of “people you encounter and interact with regularly, or are otherwise important to you even if you don't meet them very often”. The total number of individuals reported is taken as an estimate of the size of their social circle. The groups were: immediate family, other close family, distant relatives one is in contact with, other people one is in regular contact with, and other people who one feels are important to him/her. Participants wrote the number of individuals belonging to each group in an open text field. A random half of the participants were asked about their social circle before the questions about fear, and the other half after.

Note that measuring the size of social circles, or more broadly, the size of personal social networks, is a difficult methodological problem. The way a social network is defined, the questions used to ask about its size, and the ability of participants to recall their social contacts might influence the results [19]. The summation method [18] is based on the assumption that people's memories about other people are organized according to the social structure of their community and that using some common elements of social structure as prompts will improve recall [20]. Although the estimates are likely to be noisy because of various sources of error (e.g., forgetting, counting some individuals more than once), they concur with results of other methods and are reasonable proxies of the size of participants' social circles [18].

### Results

**Figure 2:**
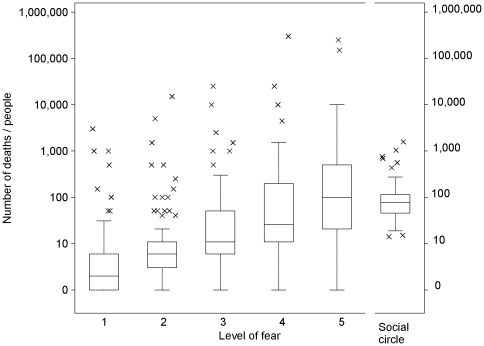
Number of deaths needed to experience different levels of fear, obtained in Experiment 6. The first five boxes show number of deaths needed to experience levels of fear from 1–5. The last box shows size of participants' social circles. Boxes represent interquartile range of estimates. Horizontal line within the boxes is the median. Whiskers cover observations that are one interquartile range away from the first and the third quartile. Crosses represent extreme outliers.

Figure 2 shows the range of participants' estimates for the number of deaths that would cause different levels of fear, as obtained in Experiment 6. The results of Experiment 5 were very similar. Median number of deaths required for fear levels 1 to 5 were in Experiment 5 (Experiment 6): 1 (1), 5 (5), 15 (10), 50 (25), and 100 (100). Even though the range of estimates was large, for the majority of participants the number of deaths that made them maximally scared was not larger than 500.

For comparison, Figure 2 shows the average social circle size of the participants. Median size of the social circle was 77 individuals. Most (95%) participants had social circles smaller than 200 individuals. These results correspond to the indicators we gathered from the previous studies^1^ [8]–[10], suggesting that the number of active contacts rarely exceeds 100 to 200 individuals. In sum, our hypothesis that dread reaches peak for risks that can kill all individuals in a typical social circle has been confirmed on the group level.

At the individual level, our results suggest that participants might have used the size of their own social circle to estimate the number of deaths needed to evoke the highest level of fear. The estimated number of deaths needed for the maximum fear was not significantly different from the number of individuals in participants' social circle (in sign test, *Z* = 0.22, *n* = 86, *P* = 0.83). Participants whose social circles were smaller than the median required fewer deaths for the maximum level of fear than those whose social circles were equal to or above the median size: *Mdn* = 40 vs. 150 deaths, respectively (in median test, χ^2^ = 4.78, *df* = 1, *P* = 0.03). On the other hand, the correlation between the size of individual social circles and the number of deaths that elicit most fear was small and not statistically significant (*rho* = 0.11, *P* = 0.30). Taken together, these results suggest that the size of own social circle affects one's estimate of the most dreaded death toll, but it is likely to be only one of several factors that affect the results at the individual level. We comment on this issue in the Discussion.

Neither the estimates of number of deaths nor those of social circle sizes were affected by participants' sex or size of the town where they lived, risk scenario, or whether the question about fear was asked before or after the question about social circle (Experiment 6). Although women and participants from smaller towns tended to require fewer deaths for maximum level of fear, this tendency was not reliably present.

## Experiments 7 and 8

An alternative explanation of the results obtained so far might have been that people do not perceive much difference between the numbers 100 and 1000. In Experiments 7 and 8, we tested this using both a context-free evaluation of numbers and a monetary loss scenario.

### Method

#### Participants

In Experiment 7, participants were 138 students at the University of Granada in Spain (18% men, age 18–36 years). Experiment 8 was conducted with *n* = 254 participants (43% men, age 18–72 years) recruited from Sozioland–a large online panel of German Internet users. Experiments 7 and 8 were conducted in April and May 2009, respectively.

#### Materials and Procedure

**As** in Experiments 1 and 2, randomly selected groups of participants in Experiment 7 were asked to rate their fear of diseases killing 10, 100, or 1000 people. After approximately 15 min of answering unrelated questions [21] randomly selected groups of participants were asked to simply rate the size of the numbers 10, 100, or 1000 without any context, using a 21-point scale ranging from 0 (“very small”) to 21 (“very large;” in the analysis we rescaled it to 11 points). In this latter context-free number size task, participants were assigned to 10, 100, or 1000 condition independently of the number they received in the former, fear of disease task. In Experiment 8, one third of participants received the fear of disease task used in Experiment 7, another third received the context-free number size task and rated the numbers using an 11-point scale, and the last third rated how upset they would feel if they lost 10, 100, or 1000 euros on an 11-point scale ranging from 0 (“not at all”) to 10 (“very much”). Within each of the thirds, randomly selected groups of participants were assigned to 10, 100, or 1000 condition.

### Results

**Figure 3:**
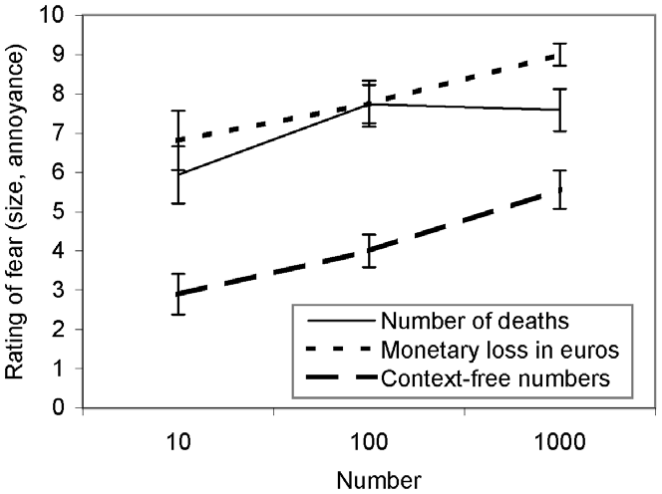
Ratings of numbers 10, 100, and 1000, in different contexts, obtained in Experiment 8. Error bars represent ±1 standard error.

In accord with our hypothesis and with previous results, participants' fear of a disease killing 100 people was larger than the fear of the same disease killing 10 people but was similar to the fear of the disease killing 1000 people (Figure 3). These results emerged consistently both in Experiment 7 and in Experiment 8. The mean difference in ratings of fear of a disease killing 10 and 100 people was 1.2±0.32, *d* = 0.51, and 1.8±0.85, *d* = 0.73, in Experiments 7 and 8, respectively, whereas the mean difference in ratings of fear of a disease killing 100 and 1000 people was −0.3±0.32, *d* = 0.15, and 0.15±0.72, *d* = 0.07, in Experiments 7 and 8, respectively.

In contrast to the results about fear of a disease, both the evaluated size of context-free numbers and the distress with monetary losses increased from 100 to 1000. The mean difference in ratings for context-free numbers 100 and 1000 was 2.0±0.90, *d* = 0.48, in Experiment 7, and 1.6±0.64, *d* = 0.89, in Experiment 8. The mean difference in ratings for being upset after losing 100 or 1000 euros was 1.3±0.64, *d* = 0.70, in Experiment 8. Taken together, these results suggest that (1) the patterns of fear ratings observed so far are not due to a general insensitivity to the difference between the numbers 100 and 1000, and (2) the effect is specific for scenarios involving people's lives rather than monetary losses or plain numbers.

## Experiment 9

On the surface, the patterns of fear found in our experiments appear to be similar to the results of studies on “psychophysical numbing” [22]–[26]. These studies have found that people's willingness to contribute to charitable causes decreases with the increasing number of people suffering. This phenomenon is thought to be a consequence of diminishing sensitivity to changes in number of deaths as the overall number of deaths becomes larger–similar to psychophysical relations observed for other physical quantities such as loudness and brightness [22]. In the present paper, we propose that the mechanism behind feelings of dread is different from psychophysical numbing. In particular, while psychophysical numbing occurs in altruistic behavior toward unfortunate people from other communities (e.g., refugees in Rwanda [22] or children in Africa [26]), the patterns in feelings of dread that we investigate originate from a potential threat to one's own community. Because the extinction of one's own community–but not that of others–may threaten one's own survival, the specific form of the relationship between fear and the size of a threatened group can be expected only for people's own communities and not for that of others. In other words, when members of people's own community are threatened, fear should increase for risks killing 10 people to those killing 100 people, and then stay approximately the same for those killing 1000 people. When other communities are threatened, fear should be on a relatively low level independently of the number of people at risk. In Experiment 9, we tested this hypothesis.

### Method

#### Participants

Ninety members of the online panel maintained by the Max Planck Institute for Human Development in Berlin, Germany participated in the study in June 2010. All participants were undergraduate students of local universities; 42% were men, with an age range of 21–36 years and an average age of 27 years.

#### Materials and Procedure

We used the disease scenario of Experiments 1, 2, 7, and 8. As in these experiments, the size of the affected group (10, 100, or 1000) was manipulated between subjects. In addition, there was a within-subject manipulation of the community affected by the disease. Each participant received three scenarios involving one group size in three different communities: Germans in Germany (i.e., participants' home country), Egyptians in Egypt (i.e., a country similar in population size but different in many other aspects; cf. Central Intelligence Agency, 2010), and German tourists currently in Egypt. The order of communities was counterbalanced. After a brief introduction, the participants read the following text: “Imagine that [Germany/Egypt/German tourists currently in Egypt] [is/are] affected by an unknown, deadly disease. Health authorities forecast that the disease will kill [10/100/1000] [people in Germany/people in Egypt/German tourists currently in Egypt] within the next week.” For each community, participants rated how afraid they would be of the disease on a scale ranging from 0 (“not at all”) to 10 (“very much”). Finally, to check the success of the community manipulation, participants rated for each community the extent of (a) empathy and (b) moral responsibility they felt toward the people affected by the disease, as well as (c) how similar they considered themselves to be to the people in the particular community, all on the same 11-point scale used for the fear assessments.

### Results

#### Manipulation checks

The type of community significantly affected participants' ratings of perceived similarity: Our participants rated themselves as most similar to people living in Germany (Mean±SE = 5.1±0.16), followed by German tourists in Egypt (3.7±0.15), and finally by people living in Egypt (3.3±0.16). In a repeated measures analysis of variance, all three communities were significantly different from each other (for difference between German tourists and people living in Egypt, *F*_1,89_ = 7.88, *P* = 0.006). The patterns for empathy and moral obligation were similar: Participants reported higher levels of empathy and moral obligation for people in Germany (5.5±0.15 and 4.2±0.16, respectively) than for either German tourists (5.2±0.15 and 3.7±0.16, respectively) or people living in Egypt (5.2±0.15 and 3.6±0.17, respectively). Because of a substantial level of variation of these ratings between participants, in the analyses that follow we include all three variables as controls.

#### Fear ratings

**Figure 4:**
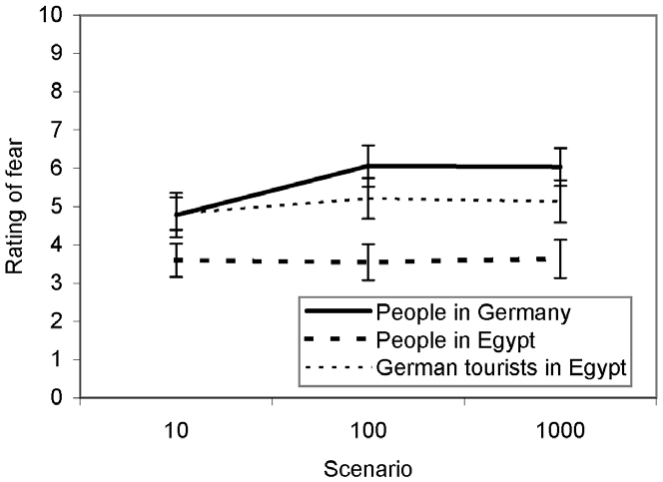
Ratings of fear killing people in different communities, obtained in Experiment 9. Error bars represent ±1 standard error.

Fear was significantly stronger when scenarios involved either people living in Germany (Mean±SE = 5.6±.32) or German tourists (5.0±.30) than when they involved people living in Egypt (3.5±.27). As Figure 4 shows, when scenarios involved people in their own community (i.e., people living in Germany), fear ratings followed the same trend we found in previous experiments: There was a significant increase in fear of risks killing 10 vs. 100 people (Mean difference±SE = 1.4±0.70, *d* = 0.53), but no difference in fear of risks killing 100 vs. 1000 people (−0.3±0.69, *d* = 0.09). In contrast, for people in other communities (i.e., German tourists in Egypt and people living in Egypt) there were no significant differences in fear for different numbers of affected individuals. These results support our argument that the dread pattern we observed is specific for people's own community and is different from psychophysical numbing, which occurs for more distant communities [22]. While death of 100 people in one's own community is dreaded more than death of 10 people, in distant communities death of 10, 100, and 1000 people provoked similar, and relatively low levels of fear.

### Discussion

In nine experiments, we found support for the hypothesis that people dread risks that threaten to wipe out the number of people corresponding to the size of a typical social circle. This pattern of fear appears consistently for several types of risk, including deadly diseases, earthquakes, and factory accidents resulting in a release of poisonous fumes into the air–suggesting that the underlying mechanism is not a specific adaptation to any particular risk (e.g., the risk of getting infected by an epidemic disease) but a more general concern about the possibility of losing one's social circle.

We hypothesized that this relationship of dread and size of typical social circle has an evolutionary origin: loosing one's group might have been deadly in ancient human history. As the extant literature [5]–[11] and our own data suggest that people tend to maintain active contact with no more than 100–150 people, threat to this number of people would be expected to evoke maximum dread. Our results are in accord with this hypothesis.

It is important to acknowledge other mechanisms that could have contributed to this pattern of results. For instance, because people seem to be cognitively adapted to maintain social contact with not more than 100–150 people [8], this group size may come to mind most naturally when trying to imagine a large group. In a related vein, people may have difficulty grasping the meaning of groups of people larger than about 100 people, as they may rarely encounter so many people in their everyday life. However, modern life seems to offer plenty of opportunities to encounter large numbers of people–from busy streets and public transportation, to sport and public events, to news about number of people using different products or being affected by war or disease. Therefore, imagining groups larger than 100–150 people is probably not very difficult in the present times.

Another possibility is that participants imagined their *own* social circle being wiped off, and therefore their expressed dread could have been a by-product of feelings of emotional attachment to its members [27], [28], rather than a product of an evolutionary adaptation to survival threats related to being alone. If this was the case, we would expect a strong correlation between the size of own social circle and the number of deaths that elicit maximum fear. However, the results of Experiment 6 suggest that the size of own social circle is only weakly related to the estimates of most dreaded death tolls, and that a general knowledge of the size of a typical social circle might be more important. This is supported by the fact that in our experiments feelings of dread were not reliably related to the size of participants' town, even though participants in smaller towns technically have higher chance to lose their social circles than participants in larger towns affected by the same risk.

The patterns of fear found in our experiments may appear to be similar to the phenomenon of psychophysical numbing [22]–[26]. However, while psychophysical numbing occurs in the context of other communities, the dread risk investigated in this study concerns threats that can plausibly affect people's own community. Indeed, in our last experiment, we found the characteristic pattern of fear ratings only for the participants' own community–in particular for compatriots currently living in the same location as the participant. As expected, fear did not change with different number of victims in an unrelated community. These results do not invalidate the phenomenon of psychophysical numbing, but suggest that it is different from the phenomenon we describe in this paper.

Regardless of whether the origin of dread is phylogenetic or ontogenetic, our results highlight the importance of people's social environments for the way they interpret and react to risks. The role of social circles in the understanding of risks has already been emphasized by Hertwig, Pachur, and their colleagues [3],[4] who showed that people use their social circles to make judgments about frequencies of health risks in the general population. In a similar vein, Olivola and Sagara [18] proposed that people's preferences for risky solutions in problems involving human fatalities are guided by the distributions of death tolls in their environment. Specifically, in environments where high death tolls are relatively rare, subjective distance between events causing intermediate number of deaths (e.g. 20 and 40) is expected to be smaller. Hence, people in these environments will be more likely to prefer risk-seeking (e.g. 50% probability that nobody will die and 50% probability that 40 will die) to risk-averse (e.g. 20 people will die) solutions to potentially deathly threats. The authors' results confirm this hypothesis: U.S. and Japanese participants, who according to the statistics rarely experience catastrophes involving large death tolls, prefer risky solutions more than Indian and Indonesian participants who experience large death tolls more often. Our findings cannot be directly compared to those of Olivola and Sagara [18] because we focus on emotional reaction to risks while they examined the effect of environmental distribution of death tolls on the curvature of individual utility functions and consequently their risk-seeking preferences. Given that it has been documented that emotion of fear affects risky choice [30], it is possible that both, fear of loosing one's community and statistical distributions of death tolls, contributed to both the present results and those of Olivola and Sagara. Further experiments could try to investigate the relative contribution of different mechanisms to the patterns we identified.

Our findings are in line with the studies conducted by Wang and his collaborators [31], [32], who showed that framing effects in risky choices involving human groups occur only when problems are presented in the context of large (e.g., with 600 or 6000 people) but not small (e.g., 6 or 60 people) group sizes, “suggesting a ‘live or die together’ small group rationality” [33]. That smaller groups have a special status when it comes to estimating risks is also echoed by Garcia-Retamero and Galesic's [34], [35] findings that medical risks are easier to understand and recall if they are presented on the basis of smaller, evolutionarily plausible groups of people.

We can exclude several possible methodological confounds of our findings. First, the results are not a consequence of a particular sample structure: We have replicated the basic patterns in different countries, with different demographic groups, and using different methodologies (i.e. laboratory and web-based experiments). Second, we believe that the results cannot be attributed to some side effect of the between-subjects design. In fact, as Birnbaum [36] showed, a more typical finding in between-subjects designs is a lack of differentiation between different numbers, or even results that violate mathematical laws (e.g., 9>221). Third, this pattern of results does not appear to be a consequence of a ceiling effect: In none of the experiments was the average fear rating of a risk that strikes 100 people larger than 8 on a scale of 0 to 10, leaving enough room for further increase. In fact, the annoyance caused by monetary losses, measured in Experiment 8, grew monotonically with the increase in monetary amounts even though it was close to the upper part of the scale from the start (see Figure 3). In addition, the pattern of results appears to be specific to scenarios involving people's lives. Evaluations of context-free numbers (Experiments 7 and 8) and monetary losses (Experiment 8) show the expected monotonic increase with the size of number.

The present results have implications for communicating risks to the general public. When the goal is to raise public awareness about a certain risk that could claim 100 or more lives, it would be beneficial to stress that the risk could kill this or larger number of people. More broadly, our results stress the importance of considering social environments when studying people's understanding of and reactions to risks.
